# The Global Business of Sport in a Brave New World: Conceptualising a Framework for Alternative Futures

**DOI:** 10.3389/fspor.2021.673178

**Published:** 2021-09-07

**Authors:** Steven J. Jackson, Marcelle C. Dawson

**Affiliations:** ^1^School of Physical Education, Sport and Exercise Sciences, University of Otago, Dunedin, New Zealand; ^2^Sociology, Gender Studies and Criminology, University of Otago and the Centre for Social Change, University of Johannesburg, Johannesburg, South Africa

**Keywords:** global sport business, sporting exceptionalism, alternative futures, sociology, sustainability, global sport

## Abstract

In July 1991, *Sports Illustrated* published a special issue featuring two articles that prognosticated about what sport would look like 10 years later. As the world entered the 21st century, *Sports Illustrated* writers, Oscar Johnson and Ron Fimrite, offered their visions of sport in the year 2001. Their analysis highlighted how a range of economic, social and technological changes in society would impact on how sport is structured, produced and consumed, but also offered insights into the future of the major professional sport leagues in North America. It has been 30 years since they publicised their views and, while technology continues to impact sport, the Covid-19 pandemic has forced the world to pause and to consider a range of deep, soul-searching questions about the nature of society, including sport. Against this background, we consider the opportunities and challenges for sport in the 21st century. The paper is divided into three sections including: (1) a reflection on the meaning, value and significance of sport including its privileged position in society, or what we refer to as “sporting exceptionalism”; (2) a brief overview of a case study that illustrates the challenges facing the global business of sport; and, (3) a framework for conceptualising alternative futures in the global business of sport, drawing on examples from women's sport.

“*Who controls the past controls the future: who controls the present controls the past*”

Orwell, *1984* (1949, p. 175)

## Introduction

In July 1991, *Sports Illustrated* published a special issue featuring two articles prognosticating about what sport would look like 10 years into the future. *Sports Illustrated* writers, Oscar Johnson and Ron Fimrite, offered their respective visions of sport in the year 2001 as the world entered the 21st century. While their forecasts were not perfect, they were prescient. For example, Johnson (1991) correctly envisaged that pay-per-view televised sport would increase, that sports gambling would be legalised and normalised, that sports fans would have increasing control over how they watched sport (including the ability to select which cameras and sound feeds they wished to receive), and that there would be increasing tensions over who owned sport leagues and broadcasting rights.

Following on from this, Fimrite (1991) expressed his apprehensions regarding the impact of new technologies, not only on sport but on the human condition more generally. For example, he shared his concerns about how the exponential growth in televised sport would reduce fans' willingness to attend live events, thus impacting not only on the business of sport, but also human interaction. Still, he readily admitted that similar fears were held about the perceived threat of earlier forms of technology, such as radio, which turned out to be largely unwarranted. However, on the matter of how new technologies may be impacting on human interaction, communication, socialisation and social bonding, Fimrite's concerns may have more merit. There are mounting and legitimate concerns about how new technologies, including social media platforms, are impacting on society (Zuboff, 2019) and young people in particular, not only in terms of their ability to relate to others and to discern reality from fiction, but also with regard to the flow on effects on well-being (Keles et al., 2020). In combination, Johnson's (1991) and Fimrite's (1991) analyses highlighted how a range of economic, social, legal and technological changes could impact on the ways in which sport is structured, produced and consumed, and how these changes, in turn, could impact on social life in the future.

Thirty years later, both sport and the global business surrounding it have not only survived a wide range of challenges, but have become one of the most powerful corporate-entertainment industries in history. Yet, some problems have endured, and new challenges and threats to global sport have emerged. Consider the following extensive and diverse list of problems facing the contemporary global business of sport:

- Integrity of Sporting Competition: this concept encompasses a range of issues that threaten the values and very essence of sport, including the use of performance enhancing drugs (PEDs), match-fixing, and the exploitation of advantages gained from the use of new technologies such as Nike's AlphaFly running shoe. Integrity is important because it is a core value of sport. Indeed—using the example of match-fixing—if the outcome of sport is in any way predetermined, the cultural practice in question is, by definition, no longer “sport” (Carpenter, 2012; Tak et al., 2017).
- Athlete welfare: This issue ranges from over-training to sexual harassment and abuse to the increasing recognition of the impact of concussion, which has been linked to Alzheimer's disease and premature death, resulting in class action lawsuits by former athletes across a range of sports (Stirling, 2009; Parent and Fortier, 2018; Kerr and Kerr, 2020).
- Discrimination: There is increasing awareness of, resistance to, and activism against, discrimination related to gender, race, indigenous rights, sexuality and disability in sport.
- Sport mega-events and human rights: The Olympics, FIFA World Cup and other sport mega-events face increasing scrutiny regarding the ethics and morality of awarding hosting rights to nation-states that have a track record of human rights violations (Lenskyj, 2000; Boykoff, 2014; Horne, 2018).
- Environment: Sports are under increasing pressure to reduce their impact on the environment, particularly activities such as motor racing (Miller, 2018) and golf that requires enormous amounts of water and the use of harmful chemicals (Wilson and Millington, 2013).
- Terrorism: Given its global visibility and significance, sporting events are not only a target of terrorist groups, but sport itself is increasingly used as a site and strategy for the recruitment of radical political extremists (Kuper, 2006; Taylor and Toohey, 2006; Giulianotti and Klauser, 2012; Spaaij, 2021).
- Corporatisation: The consolidation and monopolisation of global sport and associated media technologies and platforms, which is leading to a concentration of power and control within the hands of a decreasing number of expanding hyper-competitive, corporations. A prime example of this was the controversial 2021 proposal for a 12-team European Super League featuring the top football teams from England, Spain and Italy. The proposed new league would have consolidated the world's richest and most successful football clubs and posed a direct and immediate threat to UEFA and the current Champions League competition. Moreover, for many loyal fans the new rebel league was perceived as an attempt by rich, greedy owners to further advance their own interests at the expense of history, tradition and community.

This list of challenges is certainly not exhaustive, but hopefully it provides some insight into the scale and diversity of issues currently confronting the global elite, professional sport industry.

As per the stated aim of this special issue, we “seek to explore the impact of (changes in) economic, technological, ecological, demographic, political and social conditions of, or on, humans on how sport will be consumed in the future.” However, our focus is less upon how particular organisational structures, partnerships and technologies can simply add value to the global business of sport in the pursuit of future growth and profitability, and more on how existing problems and challenges, including the Covid-19 pandemic, offer a potential watershed moment for reflection, reappraisal and transformation. To this end, we first focus on the case of professional rugby in Aotearoa New Zealand in order to highlight problems with the global sport industry. We also consider changes that are currently underway in the realm of women's professional sport with the aim of signalling the emergence of alternative sporting futures that are more accessible, equitable and sustainable.

This essay is divided into three sections: (1) a reflection on the meaning, value and significance of sport including its privileged position in society, or what we refer to as “sporting exceptionalism”; (2) a brief overview of a case study that illustrates the challenges facing the global business of sport; and, (3) a framework for conceptualising alternative futures in the global sports industry, drawing on the example of the business of women's sport. To be clear from the outset, it is not our intention to provide an empirical analysis of the situation. Rather, we offer a critical exploration of the global business of sport through the lens of an alternative futures framework. Overall, we assert that if the current model of global sport business continues on its current trajectory, it will inevitably be plagued by some of the issues and problems noted above. Moreover, global sport will confront the limits of a business model located within an unsustainable system of consumer capitalism (Lewis, 2013). The aim of our provocation is to encourage other ways of viewing sport and its place in global society beyond the prevailing hegemonic system of consumer capitalist sport.

## Sporting Exceptionalism

The Covid-19 pandemic has had a paradoxical impact on sports fans and consumers. For some people, the global pandemic was a reminder that we should not take things, such as sport, for granted. For others, it has brought into sharp relief the fact that sport may not be the most important thing in life when considered in relation to other priorities such as family, health, work and education. Regardless of one's view, there is no denying the enduring global significance of sport, which Ulrich Beck once described as “the most important thing in the world” (Beck, 2000, p. 62). While we do not agree with Beck (and indeed we are not sure he would agree with his own statement today), there is abundant evidence that confirms the sustained social significance of sport including: the amount of media coverage it receives, the viewership numbers for major sporting spectacles, the global visibility and influence of sport celebrities and brands, the longstanding strategic use of sport in international diplomatic relations, and the emerging popularity of eSport and virtual sport, both of which are now billion dollar industries. A more recent indicator of the power and popularity of sport is the extreme effort that has gone into creating “safe” bubbles to enable the Tokyo Olympics and a wide range of professional sport leagues to operate under pandemic conditions, including: the EPL, NBA, WNBA, NFL, MLB, NHL, international football, rugby and cricket, and pro golf and tennis. Indeed, sport has emerged as a key symbolic barometer of the world's understanding, response and management of the Covid-19 pandemic. As Rowe (2020, p. 708) astutely observes: “Sport was kept constantly to the fore in debates about the “return to normality.”” The prioritisation of sport has prevailed, despite the fact that, for many epidemiological experts, exemptions for sport during the pandemic defied science, logic and morality, and put citizens at risk. The privileged position of sport during the Covid-19 pandemic leads to two interrelated questions. First, why is sport, that is, the production and consumption of a particular form of global entertainment, treated differently to almost every other type of cultural activity, including births, deaths, marriages and even societal necessities like education? And second, how does the answer to the first question enable or constrain the possibility of an alternative future for global sport business?

The answer to the first question lies, in part, at the intersection of two basic concepts that underpin all sport but in particular the broader “sports-media complex” (Jhally, 1989); that is, the overall integrated system of sport organisations, media conglomerates, corporate sponsors and sport personnel. The first concept is Coakley's (Coakley, 2015) “Great Sport Myth” (GSM), which consists of three interrelated beliefs: (1) that sport is inherently pure and good; (2) that the purity and goodness of sport is transmitted to all those who play or consume it; and (3) that sport inevitably leads to individual betterment and community development. Notably, Coakley asserts that the power of the GSM does not just emerge naturally from these beliefs, but rather that the “halo” surrounding sport is used and maintained by powerful individuals and groups to protect their interests. This is achieved by upholding particular structures and values of sport and by framing narratives about sport as being wholly in the public interest, while, in reality, many of the unintended outcomes of sport occur at the expense of the average citizen. Alluding to Antonio Gramsci's notion of hegemony and the role of culture in winning consent, Coakley (2021, p. 403) asserts that, “It's as if ruling elites had read Gramsci and concluded that sport, more than other civil institutions today, appeals to popular tastes in ways that make people gullible and subject to political manipulation and control.” This is not to suggest that people are cultural dupes, but rather that those in positions of power have increasingly more complex, pervasive, and immediate control over media technologies, platforms, content and narratives through which to maintain hegemony.

The second concept is what we refer to as “sporting exceptionalism”; a notion that combines and reinforces the effects of the GSM and is embedded in a set of assumptions that reproduces a generalised belief that the history, structure and values of sport are unique, positive and universally accepted compared to other cultural practices and institutions. As a consequence, the dominant representation of sport—and the socio-economic system within which it is located—are seldom questioned, and alternatives rarely considered. Consider two basic examples that illustrate the exceptionalism of sport. First, sport is a law unto itself given that, by and large, it self-regulates and self-polices across a wide range of serious, often criminal, matters including athlete maltreatment, violence, doping, match-fixing and corruption (Stirling, 2009; Kihl et al., 2017; Tak et al., 2018; Young, 2019). Second, sporting exceptionalism is confirmed by its privileged treatment in the face of a global pandemic where, professional sport, was recognised “as an essential industry in which athletes, audiences and stadia were represented as the symbolic equivalents, respectively, of health professionals, patients, and hospitals” (Rowe, 2020, p. 708).

In combination, the concepts of the Great Sport Myth and sporting exceptionalism help to explain the ideological, cultural, economic and political power of sport, and how and why it is rarely challenged. Indeed, these concepts sharpen our understanding of “uber-sport,” a heuristic device coined by Andrews (2019) to explain a phenomenon wherein “an amalgam of corporate capitalism, consumer culture, neoliberalism, and nationalism frames the constitution and experience of professional sport as a mass entertainment product” (Andrews, 2019, p. 8). While Andrews' insights focused specifically on the US context, we suggest that the notion of “uber-sport” can be employed in other contexts too. We turn now to the case of professional rugby in Aotearoa New Zealand in order to highlight some of the fundamental problems facing the contemporary global sport industry. Our assertion is that the current model of sport is flawed, and that if we aspire for a more accessible, more equitable, and sustainable version, then we need to consider some bold alternative futures not only in the realm of sport, but also in society more generally.

## Case Study: New Zealand Rugby

Rugby is neither the largest nor most popular sport in the world, but it does offer some unique insights with respect to understanding the challenges facing the future of global sport business. For example, although it has a long history dating back to the 1800s, rugby did not officially turn professional until 1995 and, as such, provides a useful case study for understanding the development and challenges of a fairly new global professional sport. Here, we focus on the case of New Zealand rugby for a range of reasons: rugby is often unquestionably considered to be the national sport [indeed, Fougere (1989), asserted that it predated the actual formation of the nation-state itself]; the country is geographically remote and has a relatively small population (approximately five million) and economy; and, the men's national team, the All Blacks, are arguably the most successful sporting franchise in history with a 75 per cent win record over more than a century.

We begin by putting the business of rugby, in general, and the All Blacks as the primary generator of revenue for the corporate body that oversees the national sport, New Zealand Rugby (NZR), in particular, in context. In the early 1990s, two rival media moguls, Kerry Packer and Rupert Murdoch were embroiled in what FitzSimons (1996) described as the “Rugby War.” In the lead-up to the 1995 Rugby World Cup, Kerry Packer's World Rugby Corporation (WRC) aggressively pursued broadcast deals and lucrative contracts to attract the best rugby players in New Zealand, Australia and South Africa. In response, and in an effort to ward off Packer's master plan, the New Zealand Rugby Football Union (NZRFU), as it was then known (later becoming the NZRU and eventually rebranding simply as the NZR), in conjunction with the Australian and South African Rugby Unions formed (SANZAR, which became SANZAAR in 2016 with the addition of the Argentina Rugby Union), signed a 10-year, $800-plus million-dollar world-wide broadcast deal with Rupert Murdoch's News Limited. The deal, which provided millions of dollars to the NZRFU, was a major turning point for the sport of rugby generally and New Zealand's national game in particular. Not only did the broadcast deal provide crucial funding to retain top young players, it also contributed to grassroots development. Unsurprisingly, the News Limited broadcast deal, which operated in New Zealand under the Sky Network Television network, soon attracted sponsorship opportunities. In 1999, global sportswear company Adidas became an official sponsor of New Zealand Rugby offering one of its biggest team contracts at that time, estimated at more than $US100 million dollars over five years. The agreement represented a strategic decision by Adidas to: (a) capitalise on a new sport/team that was likely to succeed outside Europe and North America, and (b) harness the major marketing opportunity that media coverage of rugby provided (Adidas thinks big over All Blacks, 1999, p. 11). As a global sporting commodity (and the most powerful within world rugby), the All Blacks became a very attractive “brand.” Indeed, Howard Greive, the Saatchi and Saatchi advertising executive, who was responsible for producing the first new Adidas advertising campaign, argued that: “the All Blacks can deliver something to the [Adidas'] brand that no other team or individual can in sport” (Anon, 1999, p. 22–23). Evidence of the enduring value of the All Blacks to the company is the fact that Adidas has remained a loyal sponsor, with its most recent contract, which runs from 2017 to 2023, estimated at $US10 million per year.

In many ways the Sky TV and Adidas deals saved or, perhaps more accurately, sustained NZR. Yet, this major injection of funding was in sufficient in the long run. Moreover, it was not viewed positively by everyone. For example, former All Black and New Zealand diplomat, Laidlaw (1999, p. 177–178) warned in 1999 that:

There is a real danger that control of the game in New Zealand will steadily be wrested away from New Zealand hands. … It is the bottom line of the national personality that is at stake and we are in danger of letting McWorld have it for a few pieces of silver. [However] [t]he massive deals that have been done with major sponsors were the only way of preserving that sovereignty.

Laidlaw's comments capture the paradox of contemporary global sport business; that is, to secure enough funding to survive in the future, one might need to negotiate a deal that relinquishes some control and ownership to foreign interests. This conundrum has repeated over the past two decades and is evident in the ongoing challenges faced by New Zealand as a nation and the All Blacks as a sport franchise operating within the new global economy of sport (Scherer and Jackson, 2007, 2010, 2013). For example, in 2012 Adidas was joined by another major global corporate sponsor, American Insurance Group (AIG). AIG is a company with more than 90 years of history that boasts an annual revenue in excess of $US60 billion, 88 million clients in 130 countries and almost 50,000 employees. Aside from the unique features that the All Blacks offered corporate sponsors (Jackson et al., 2001), AIG's interest stemmed from another major global sporting development. In 2009, the International Olympic Committee announced that it was adding Sevens Rugby to the 2016 Olympics. As a consequence, there was an acceleration of investment in and global growth of the sport in nations seeking to secure one or more of the six new medals on offer (Women's and Men's gold, silver and bronze). Moreover, as the largest sporting spectacle on earth, the Olympics provided unprecedented brand exposure for corporate sponsors. Thus, AIG signed a 9-year deal (2012–2021) estimated at $US14 million per year. In return for this large investment, AIG made some major demands including prime logo placement in the centre of the All Blacks jersey; a space that had been left blank for more than 100 years and which some would consider sacred. Moreover, they negotiated the sponsorship to include the rebranding of all New Zealand national teams: the All Blacks, the Black Ferns, the Māori All Blacks, Men's and Women's Sevens teams and the Under-20 Men's team. The case of Olympic Sevens Rugby and subsequent AIG sponsorship highlights not only the power of global sport organisations, such as the IOC, to determine what is defined as legitimate sport, but also their ability to influence nation-state investment in targeted sport development on one hand, and the links to the sport-media complex (including broadcast rights and corporate sponsorship) on the other.

Notably, not unlike when Adidas entered the New Zealand sporting landscape in 1999, the new AIG sponsorship deal raised immediate concerns from a range of stakeholders. For example, while Adidas had reservations about how AIG's presence might clash with their own brand visibility, New Zealand Rugby was apprehensive about the potential demands and loyalty of the new American sponsor. As if echoing Chris Laidlaw's lament from 1999, Steve Tew, the former CEO of NZR, highlighted the challenges of being a small nation-state within the global sport business economy:

We are in a challenging time in this world we live in. … We have a business that has roughly $100 million turnover a year—it needs to be significantly more than that if we're going to survive, if we're going to grow the game at the community level and if we're going to retain players. It's a challenge for us, and we need some money. (APNZ, 2012).

The AIG deal highlights the ambivalence that NZR, and the wider New Zealand public, hold towards the increasing commercialisation of the national game. Despite the initial misgivings of many, the AIG sponsorship no doubt helped buoy the All Blacks over the next decade, and the necessity of such deals has come to be widely accepted as essential to the game's survival. The recent, and unexpected, announcement that AIG's sponsorship would end in 2021 has raised major financial concerns and revealed the game's reliance on corporate backers.

Yet, beyond the impending loss of the AIG sponsorship, NZR has been facing a raft of equally significant problems including: a dramatic decline in participation rates amongst young males, revelations of rising rates of head injuries resulting in the threat of lawsuits from former players, a hypermasculine sporting subculture that has been under increasing public scrutiny, and a growing number of players leaving New Zealand for lucrative overseas contracts.

However, perhaps the biggest threat to NZR (and other non-European rugby nations) has been the failure of World Rugby (formerly the International Rugby Board, IRB)—the international body responsible for governing and developing the sport—to develop a sustainable model for the future. Many nations have become increasingly vocal about the need for a new global competition that would not only elevate international interest and attract additional media and corporate investment, but would also promote the global growth of the game into new regions, facilitate a more equitable distribution of funding and help improve player welfare by balancing competition schedules. Unfortunately, as it is currently structured and governed, World Rugby is dominated by the rugby unions of the Six Nations (England, Wales, Scotland, Ireland, France and Italy) each of which is allocated three votes, giving them a clear advantage with respect to decision-making. Highlighting the severity and urgency of the problem in 2020 when the effects of Covid-19 were beginning to be realised, NZR chairman, Brent Impey, issued a stark warning about World Rugby's complacency: “If these guys don't get on and make change, if it's four more years of the same, we'll [NZR] be gone” (Napier, 2020). Confirming the reality of the challenge, the 2020 NZR annual report revealed a $US24 million dollar loss.

Despite the concerns about NZR's financial stability there have been some recent signs of optimism. Over the past year, it has explored potential partnerships with overseas investors and, in February 2021, news broke that NZR was in advanced discussions with Silverlake, a Silicon Valley-based technology investment company with interests in Twitter, Dell, Airbnb, Broadcom and Alibaba (Keall, 2021). Preliminary reports indicate that Silverlake has offered $US323 million in exchange for a 15% share of NZR's commercial rights (Napier, 2021). Part of the appeal of Silverlake for the NZR is that it has a track record within global sport business with direct or indirect investments in UFC (through holding company Endeavour), Madison Square Garden Company (which owns the NBA's New York Knicks and the NHL's New York Rangers) and City Football Group, whose flagship team is Manchester City. In addition, Silverlake offers financial strength and stability with an estimated US$83 billion in assets, while its investment portfolio generates more than $US180 billion revenue annually (Napier, 2021). However, a key question is why would a successful American technology investment company be interested in the sport of rugby, particularly in a small market nation like New Zealand? The answer may be multidimensional but, alas, not necessarily complicated. While NZ itself may be a small market, NZR's commercial rights, owing largely to the powerful All Blacks brand, is currently valued at US$2.2 billion. This value is quite incredible given both the modest profile of rugby as a global sport and the size of New Zealand's market, and it stacks up well when compared to elite franchises such as the LA Lakers valued at US$4.4b and Manchester United, ranked third amongst the world's most valuable football brands, worth US$3.8b. In all likelihood, Silverlake's ability to use their extensive media technologies and networks to leverage these commercial rights is at the heart of their bid for rugby in Aotearoa New Zealand.

Some view the Silverlake offer as the saviour of NZR and a turning point in its history. Others, however, are not only sceptical, but fear that the deal will render NZR at the mercy of a global corporate entity whose loyalty is unproven, whose values and objectives may not align with those of rugby supporters, and who has the potential to sell, trade or otherwise destroy the very fabric of the national sport in the future. Many of the fears stem partly from NZR's broad roles and responsibilities beyond the professional game. As New Zealand Herald sports journalist, Dylan Cleaver (2021), has noted, there are a couple of unique features of NZR's organisational functions that may distinguish it from other global sport franchises. First, while most professional sport franchises are privately-owned businesses with a focus on delivering profits for their owners and shareholders, NZR “has an obligation to act in the best interests of its key stakeholders, the most important being its 26 [provincial] unions. Those 26 unions, in turn, are charged with running not just representative teams, but fostering the growth of community rugby” (Dylan Cleaver, 2021). In short, in contrast to other professional sport organisations, NZR has broader social responsibilities with respect to supporting grassroots sport.

Another local factor complicating the Silverlake bid relates to the fact that in 2019, NZR entered into a unique partnership with its local media broadcaster, Sky TV. While some details are confidential, it is estimated that Sky TV paid $US340 million for the broadcast rights rugby in New Zealand from 2020–2025. Highlighting both the perceived value of rugby as an entertainment commodity and the intense competition for media content, this figure is almost double the amount Sky TV paid for these rights in its previous contract. However, there is one other distinctive feature of the NZR-Sky TV deal; that is, as part of the agreement, NZR has taken a five percent equity stake in Sky TV, which, in 2019 was worth about $20m (Herald, 2019). Given the longstanding partnership, which dates back to 1995, and the rather fragile state of NZR finances, the deal seemed both logical and mutually beneficial. However, just over a year later, the value of NZR's shares in Sky TV had dropped from $US14m to < $US2.7m largely due to the media company's continued decline in the face of competition from other broadcasters. The NZR-Sky TV deal highlights both the unique and vulnerable nature of the sport-media complex and associated contracts within a small market like Aotearoa New Zealand. Beyond the risk of being an investor in Sky TV, NZR may now have to carefully navigate negotiations with Silverlake who, as an investor with its own broadcast and streaming technologies, expertise, networks and aspirations, will seek to maximise the value of All Black test matches both locally and globally. While there are many unanswered questions, one immediate red flag for NZR might be the fact that the company donated more than US$4m to American political interests over the previous four-year electoral cycle, most of it to Donald Trump and the Republican Party (Keall, 2021). Moreover, NZR will need to be cognisant of the fact that if the current proposal proceeds it will not be able to control whether, when or to whom Silverlake may want to sell its 15% stake in the future.

The case and fate of NZR highlights one example of the challenges facing global sport business and serves as a timely reminder that the world is more interconnected than ever before. As such, “no sporting nation is an island even if, geographically, it is one” (Rowe, 2020, p. 706). Yet, the situation is even more complicated because, despite the potential positives associated with a deal with Silverlake, NZR is already behind in the global media stakes game. Consider the fact that a rival private equity firm, Luxembourg-based CVC, negotiated major investment and media deals with Six Nations Rugby. As of 2021, CVC has a “long-term strategic partnership” which will see it gain a 14.3% share in Six Nations Rugby, a 27% share of Premiership Rugby, the body that oversees the top rugby division in England, and a 28% stake in the Pro14, the major European cross-border rugby competition (Lewis, 2021). Consequently, inasmuch as Silverlake's investment may help to sustain New Zealand Rugby in the short term, the reality is that the combined political and economic power of Six Nations Rugby—in terms of both voting dominance within World Rugby and the enormous media and investment deals such as those of CVC—will continue to provide significant advantages over its Southern counterparts and will likely see those in the North remain the key powerbrokers in global rugby.

The case of professional rugby provides an example of some of the challenges facing one particular sport in the contemporary world of global sport business. Here we have outlined a scenario of the challenges and opportunities within a “business as usual” scenario. Regardless of the debates about Silverlake or any other foreign investors in New Zealand rugby, all of these proposals are based on the existing consumer capitalist model of sport rather than any type of fundamental, transformational change^1^. We contend that it is prudent, and indeed essential, for those involved directly in the global business of sport as well as humanity at large, to consider alternatives to the current structure and ideological underpinnings of contemporary sport. To this end, the next section offers a conceptual framework emerging out of the literature on alternative futures, with the aim of stimulating new ways of thinking about global sport business.

### Alternative Futures: A Conceptual Framework

If legacies are about story-telling, how would we like the story of the global sport industry in the early part of the 21st century to be told to those learning about it in 22nd? We could capitulate to the “TINA” (there is no alternative) narrative and accept “uber-sport” (Andrews, 2019) as “common sense” (Gramsci, 1971), thereby giving ourselves license to rehash the same stock stories that reinforce structures of power and privilege (Bell, 2019). Stock stories, however, deny the potential of collective human agency to transform the world. Another option is to accept Marx's invitation not only to interpret the world, but also to change it (Marx, 2002), which requires us to produce counter-stories (Bell, 2019); an alternative way of thinking about the global business of sport. We choose the latter.

The conceptual building blocks for our counterhegemonic project within the business of sport are inspired by Dawson's (2016) three-step framework for alternative futures and Jarvie, Thornton and Mackie's (Jarvie et al., 2018) insights into the potential of sport as a ‘resource of hope'. Dawson's (2016) quest to find “sociological alternatives” begins with the seemingly obvious task of identifying a social problem. Likewise, Jarvie et al. (2018) encourage us to ask: “What is going on out there in the world of sport?” Of course, pinpointing the problem is no simple matter. For example, we may not all agree on *what* the problem is, or *why* it has come to be defined or experienced as a problem, *who* has the power to define something as a problem, or *for whom* it is perceived as a problem in the first place. Thus, embedded in this first phase of building alternative futures lies the sociological duty of exposing and disrupting power relations. Such an exercise is inherent in Jarvie's (2018) second task, namely providing an analysis and explanation.

Once the problem has been identified and its dimensions analysed, both Dawson (2016) and Jarvie et al. (2018) suggest proposing an alternative. In so doing, we are encouraged to move beyond a politics of grievance towards a call and/or plan for collective action. It is at this stage that we are tasked with mapping out a vision for the future, not only in terms of nature/direction and scale, but also in terms of duration (i.e., is the alternative for now or forever?). This step harnesses the uniquely human ability to imagine and act on that imagination, or to desire and create, which offers a more humanist interpretation of the processes of consumption and production inherent in crude, reductionistic accounts of the economy (Graeber, 2007). In other words, formulating alternatives for the future is a fundamentally human task. It is not up to the “invisible hand” of the market to decide what humans should want or how they should live in the future. Jarvie et al. (2018) extend this step by asking us to consider what our own individual role or contribution to change would entail. In other words, this second stage of formulating alternative futures is ultimately about collective and individual agency.

Perhaps the most crucial step in building an alternative society is the last one, which entails justifying how the proposed alternative addresses the problem identified at the start (Dawson, 2016, p. 3). Here, we have the opportunity to consider possible unintended consequences and to engage in prolepsis, a literary technique used to peek ahead to events that may only come to fruition later (Yates, 2015). It is a clever stratagem that blurs the line between present and future and allows one to resolve the puzzle of knowing then what one knows now. Prolepsis raises the possibility that one might behave differently if one could see into the future. In a practical sense, this stage affords an opportunity to play devil's advocate and map out various responses or plans of action, depending on potential/hypothetical criticisms against one's proposed alternative.

To demonstrate the value and broader implications of Dawson's (2016) and Jarvie's (2018) frameworks for alternative sporting futures, we turn our attention to the current landscape of women's professional sport. Specifically, we briefly consider the status and challenges of women's professional sport in relation to the three steps: (1) identifying the problem and analysing its dimensions; (2) proposing an alternative with consideration for our individual and collective agency; and, (3) explaining how the proposed alternative addresses the original problem along with any potential unintended consequences. One of the enduring challenges facing women's professional sport is inequity in terms of access, resourcing, media coverage, sponsorship and salaries. Without doubt, the evidence concerning the barriers to and inequities of access, resourcing, media representation and sponsorship of women's sport is overwhelming. Yet, with respect to the first step of creating alternative futures, not everyone would identify all of these issues as systemic problems but may instead regard them in reductionist terms of supply and demand. That is, within a market-driven system, critics have noted that those who own and hold the power to produce sport, tend to argue that the consumer decides what content they will watch, what they are willing to pay, and which sponsors they are willing to support (Cooky et al., 2013; Kane et al., 2013; Bruce, 2016). The market forces perspective enables sport organisations to defend greater levels of investment in men's sport, enables sports media to defend the overrepresentation of men's sport across a broad range of communication platforms, and enables sports brands to justify significantly more financing and market profile of men's celebrity athletes, teams and events. In short, for those who adopt a market perspective, the central issue is not gender equality but simply part of the realities of global sport business. Within this scenario, and drawing upon the alternative futures framework outlined above, one logical step would be to try and develop the market for women's professional sport. Indeed, this process has been unfolding in different sports and at different rates over the past three decades. Yet, challenges and barriers to the advancement of women's professional sport remain, because existing alternatives have failed to address the systemic roots of inequality (Fink, 2015; Cooky and Messner, 2018). Next, we discuss the implications of proposing alternatives and exercising agency for women's sport.

While Covid-19 has impacted on all sport, there is evidence to suggest that it has had a particularly negative impact on girls' and women's sport. In part this is due to the pre-existing, historical inequities in support, profile, funding and resources. Of course, these constraints were not caused by the global pandemic but were, instead, exacerbated by it, owing to the prioritisation of men's sport and its privileged location within the sport-media complex. That is, since men's sport is currently the key driver of the global sport economy, it was viewed and treated as an “essential” industry by governments, broadcasters and corporate sponsors. What is perhaps most unfortunate is the fact that there were pockets of women's sport that were beginning to thrive with respect to participation and spectator numbers, media coverage and corporate sponsorship (Willson et al., 2018; Toffoletti and Palmer, 2019; Pavlidis et al., 2020). On this note there is some optimism. For example, Rebecca Sowden—a former New Zealand football (soccer) international representative who now operates her own marketing and sponsorship company, Team Heroine^2^– offers five key predictions for the future of women's sport including: (1) more player-led content, (2) more collective action, (3) different ownership models, (4) individual athlete and team activism, and (5) a rise in long-term sponsorship deals. In and of themselves, these predictions are neither naïve, nor impossible, but it is important to locate them within a socio-historical context that has long privileged male sport. Notably, some of Sowden's predictions constitute aspects of the second step in the alternative futures framework; that is, proposing and enacting alternatives. Moreover, it is important to emphasise that many of the intended benefits of these initiatives are beginning to be realised. For example, the expansion of media networks that are seeking novel content has offered new opportunities for women's sport. Likewise, social media has created new spaces for promoting events and sharing information about women's sport (Thorpe et al., 2017).

With respect to collective action, consider “The Fan Project”^3^, which was launched on February 3, 2021 as part of the USA's National Girls & Women in Sports Day. The aim of the Fan Project is to increase the visibility of women's sport via media coverage and lobbying of corporate sponsors. According to Sowden, The Fan Project “is a good example of collective action. … All the top women's sports leagues have basically joined forces and are asking fans to download their Facebook and Twitter data to them so they can shape conversations, and take it to the networks and sponsors to show how much interest there is” (Stanley, 2021). The Fan Project was ostensibly prompted by a “problem” or shortcoming highlighted by a male broadcasting executive, who claimed, “If we had more data that proved fans will watch women's sports we'd give the games a lot more air time” (The Fan Project, 2021a). The response by The Fan Project was to give executives and broadcasters precisely what they asked for. In terms of justifying why the proposed alternative addresses the problem—Dawson's third step—The Fan Project explains its vision for the future as follows: “The more visible that women's sports are, the greater their power is to inspire the next generation. With your data, we'll unlock the power to push executives to increase coverage of women's sports to 10% of all sports coverage in 10 months' time. Only when women's sports are seen can they inspire” (The Fan Project, 2021a). The Fan Project regards the validation of fans' interest in and desire for more women's sports, as a step towards achieving more gender parity in sports. They claim that “[e]quality in sports isn't just right—it's smart business” (The Fan Project, 2021b). We are, however, less optimistic that the alternative proposed by the Fan Project will have a marked effect on the future of the global business of sport. We will return to this point later on.

The Fan Project is just one example of an attempt to transform women's sport. With respect to new ownership models within sport there are also positive signs. For example, tennis star Naomi Osaka has recently become a co-owner of the North Carolina Courage, defending champions of USA's National Women's Soccer League (NWSL). Similarly, a new NWSL franchise, Angel City FC, will enter the competition in 2022. This new team is co-owned by former USA Women's football star Mia Hamm, and others including, actresses Natalie Portman and Eva Longoria. What these examples highlight is a potential cultural shift in how women's sport is structured, produced, represented and consumed. Given that women make up 50% of the world's population and have increasing political, economic and cultural power (at least in the West), there is an opportunity to use their collective influence (and consumer power) to enact change. However, cautionary notes remain, and in this final section of the discussion, we turn our attention to the third step in the alternative futures framework, namely justifying how the proposed alternative addresses the original problem and identifying any potential unintended consequences.

Given the enduring challenges faced by women's professional sport, it would be expedient to accept that any increase in awareness, access, support and investment in women's sport is positive. On this account, women's sport has made significant advances including: increasing power through positions on the boards of major sport organisations and media companies, and increasing visibility with respect to improved quantity and quality of media coverage, which has, in turn, attracted more corporate sponsorship. Moreover, there are increasing opportunities for young girls to participate in sports that had previously been defined or perceived as “male” sports with participation rates soaring in codes such as soccer, ice hockey and rugby. However, heeding Dawson's (2016) advice, it is important that we engage in prolepsis as a way of envisioning the future in order to anticipate potential unintentional consequences. Here, it is important to acknowledge that as things currently stand, women's professional sport operates within the same consumer capitalist, market-driven, system as men's sport. On one hand, the men's model of professional sport provides society with large scale spectacles, such as the Super Bowl or Olympics, where we can witness elite athletes continually pushing the boundaries of what the human body can achieve. Moreover, such events and the celebrity athletes and teams that are showcased provide role models, a sense of community and even nationalism. Yet, there are negative counterparts to all of these positive attributes of sport including many of those listed at the beginning of this paper: doping, exploitation, corruption, ethnonationalism, diplomatic conflict, and violence and injury.

If we take the example of The Fan Project's proposed alternative of encouraging supporters to share their social media data with the aim of encouraging network executives and sponsors to invest more and offer more equitable resourcing to women's sport, there is no indication that this shift will address the deep-rooted problems currently facing the contemporary global business of sport. The Fan Project's alternative, albeit commendable, does not propose a radical break from the status quo. Rather, it attempts to cast women's sport in the same mould as men's sport and, in so doing, reinforces consumer capitalism. It is our contention that gender inequality (and other forms of discrimination and oppression) are rooted in the consumer capitalist model that dominates the global business of sport, and it is not the case that the myriad challenges within women's sport will simply be resolved if it were afforded the same privileges as men's sport. While the alternative proposed by The Fan Project may lead to some reforms in the short-term, we argue that its justification for the alternative falls short of Dawson's (2016) requirement that in order for a proposed change to engender a viable social alternative, it needs to offer a convincing improvement on the status quo. If it fails to do so, it is, at best, change for change's sake. At worst, it reinforces the systemic fault lines that give rise to social problems in the first place.

Peering into the future, we might ask whether the advancement of women's professional sport within the current model is the best way forward? Is there a risk that if elite women's sport follows the men's model, it will face the same problems, but have less resilience given its shorter history and longevity? If so, what alternative future for women's sport might be envisioned? Thinking about the future is not just about planning ahead. It requires familiarity with our immediate realities (i.e., the present), but also—crucially—with the past. Awareness of all three dimensions of time allows us to ascertain whether what might eventuate in the future is an altogether new occurrence, that is, a rupture from the past, or whether it is simply a contemporary occurrence of something that has happened previously. Without this distinction, it becomes easy to confuse what is “now” with what is “new.” In addition to a robust understanding of past and present, crafting an alternative future requires a radical imagination; an ability to envision *and enact* a social reality that has yet to emerge. Our discussion began with a nod to Orwell, and we would like to end by extending his insights even further by suggesting that we need to concern ourselves with making the future in the present; producing in the here and now the world that will be inhabited tomorrow and remembered in the distant future. Arguably, it is in all our interests to think about the kind of world we desire in the future, based on what we know about the past, and then begin, in the present, to create it. By extending Matt Dawson's (2016) futures framework to include concepts such as ‘absences and emergences' (Santos, 2006), “alternative conceptions of time” (Marcuse, 1969; Toffler, 1970; Winter, 2020) and “prefiguration” (Boggs, 1977; Maeckelbergh, 2011; Yates, 2015), we suggest that crafting alternative futures is much more than an aspirational task. We agree with (Schultz, 2016, p. 15) that, “[t]he future is not just happening but it is made, and there are choices,” and, drawing on examples from the world of sport, we argue that building alternative futures is a highly pragmatic and, indeed, urgent undertaking that requires a collective and coordinated effort.

### Conclusion

This analysis has highlighted the unique and powerful status of sport in society and within the brave new world of global business. The power of sport emerges, in part, from the Great Sport Myth (Coakley, 2015) and the concept of “sporting exceptionalism” which, in combination, creates a halo around sport that is used and maintained by powerful individuals and groups to protect their interests. This is achieved by upholding particular structures and values of sport and by framing narratives about sport as being in the public interest but ultimately at the expense of the average citizen. Using the case study of professional rugby and its location within the wider context and structures of World Rugby, we highlighted some of the opportunities and challenges facing one particular country, New Zealand, as a small nation located on the peripheries of both geography and political power. We then combined Dawson's “sociological alternatives” and Jarvie et al's notion of sport as a resource for the future, to offer a framework to think about the future of global sport business. Here, we used the example of women's professional sport to illustrate his three key steps for an alternative future, while also signalling some of the risks associated with following the hegemonic male model of consumer capitalist sport with all of its problems and limitations.

Conceptualising alternative futures is challenging because it requires us to consider possibilities that are not yet known. Indeed, the more dominant or hegemonic any existing social system, including sport, the more difficult it is to consider, let alone, enact an alternative future. Yet, there are lessons to be learned and enormous potential value in applying the concept of alternative futures and contemplating how it might contribute to new structures, policies, and programmes that will positively transform global sport business. Future research in this area may wish to focus on some of the key challenges identified in this analysis in order to explore how an alternative futures framework might help reconceptualise the problem and thereby contribute to new solutions.

## Data Availability Statement

The original contributions presented in the study are included in the article/supplementary material, further inquiries can be directed to the corresponding author.

## Author Contributions

All authors listed have made a substantial, direct and intellectual contribution to the work, and approved it for publication.

## Conflict of Interest

The authors declare that the research was conducted in the absence of any commercial or financial relationships that could be construed as a potential conflict of interest.

## Publisher's Note

All claims expressed in this article are solely those of the authors and do not necessarily represent those of their affiliated organizations, or those of the publisher, the editors and the reviewers. Any product that may be evaluated in this article, or claim that may be made by its manufacturer, is not guaranteed or endorsed by the publisher.

## Footnotes

^1^There have been a number of subsequent and alternative proposals to the Silverlake deal tabled. For example, the New Zealand Rugby Players Association offered suggestions ranging from the possibility of a public share float that would enable everyday citizens the opportunity to invest in the commercial arm of the NZR. The concept of partial public ownership of professional sport franchises does exist and offers one potential alternative for the future. However, given that the NZR signed a controversial 6-year sponsorship deal with UK-based petro-chemical company, Ineos, for an estimated $US3.4-5.6 million per year in July, 2021, suggests that it is not yet capable or ready for transformational change.

^2^ http://www.teamheroine.com

^3^ http://www.thefanproject.co

## References

[B1] Adidas thinks big over All Blacks (1999, July 16). National Business Review, p. 11.

[B2] Andrews D. (2019). Making Sport Great Again: The Uber-Sport Assemblage, Neoliberalism, and the Trump Conjuncture. London: Palgrave Pivot. 10.1007/978-3-030-15002-0

[B3] Anon (1999). Primal Team. AdMedia 14, 22–23.

[B4] APNZ (2012). Sponsor's Logo Helps Black Jersey to Survive. New Zealand Herald. Available online at: https://www.nzherald.co.nz/nz/sponsors-logo-helps-black-jersey-to-survive/5PQ67JNWNQHFMLXUBAABUDECLE/ (accessed January 21, 2021).

[B5] Beck U. (2000). The Brave New World of Work. Cambridge: Polity Press.

[B6] Bell L. A. (2019). Storytelling for Social Justice: Connecting Narrative and the Arts in Antiracist Teaching (2nd Edn.). London: Routledge. 10.4324/9780203852231

[B7] Boggs C. (1977). Marxism, prefigurative communism and the problem of workers' control. Radical Am. 6, 99–122.

[B8] Boykoff J. (2014). Activism and the Olympics: Dissent at the Games in Vancouver and London. New Brunswick, NJ: Rutgers University Press.

[B9] Bruce T. (2016). New rules for New Times: sportswomen and media representation in the third wave. Sex Roles 74, 361–376. 10.1007/s11199-015-0497-6

[B10] Carpenter K. (2012). Match-fixing - the biggest threat to sport in the 21st century? Int. Sports Law Rev. 2, 13–24.

[B11] Coakley J. (2015). Assessing the sociology of sport: on cultural sensibilities and the great sport myth. Int. Rev. Sociol. Sport 50, 402–406. 10.1177/1012690214538864

[B12] Coakley J. (2021). Sports in Society: Issues and Controversies, 13th Edn. New York, NY: McGraw-Hill Higher Education.

[B13] Cooky C. Messner M. Hextrum R. (2013). https://journals.sagepub.com/doi/abs/10.1177/2167479513476947 Women play sport, but not on TV: a longitudinal study of televised news media. Commun. Sport 1, 203–230. 10.1177/2167479513476947

[B14] Cooky C. Messner M. A. (2018). No Slam Dunk: Gender, Sport and The Unevenness of Social Change. New Brunswick, NJ: Rutgers University Press. 10.2307/j.ctt1t6p7fx

[B15] Dawson M. (2016). Social Theory for Alternative Societies. London; New York, NY: Palgrave. 10.1007/978-1-137-33734-4

[B16] Dylan Cleaver D. (2021). Rugby: Dylan Cleaver - Inside New Zealand Rugby's greatest gamble. New Zealand Herald. Available online at: https://www.nzherald.co.nz/sport/rugby-dylan-cleaver-inside-new-zealand-rugbys-greatest-gamble/LYW2TICC3U5JE3C2F3HSDLP43U/ (accessed February 3, 2021).

[B17] Fimrite R. (1991). What if they held a sporting event and nobody came? The author decries a future in which television replaces the camaraderie of the crowd. Sports Illust. 75, 51–54.

[B18] Fink J. S. (2015). Female athletes, women's sport, and the sport media commercial complex: Have we really “come a long way, baby”? Sport Manage. Rev. 18, 331–342. 10.1016/j.smr.2014.05.001

[B19] FitzSimons P. (1996). The Rugby War. Sydney: HarperCollins.

[B20] Fougere G. (1989). “Sport, culture and identity: the case of rugby football,” in Culture and Identity in New Zealand, eds Novitz D. Willmott B. (Wellington: GP Books), 110–122.

[B21] Giulianotti R. Klauser F. (2012). Sport mega-events and ‘terrorism': a critical analysis. Int. Rev. Sociol. Sport 47, 307–323. 10.1177/1012690211433454

[B22] Graeber D. (2007). Revolution in Reverse. The Anarchist Library. Available online at: https://archive.org/details/al_David_Graeber_Revolution_in_Reverse_a4/page/n1/mode/2up (accessed February 15, 2021).

[B23] Gramsci A. (1971). Selections From the Prison Notebooks of Antonio Gramsci. Edited and Transl. by Q. Hoare and G. Nowell Smith. London: Lawrence and Wishart.

[B24] Herald N. Z. (2019). ‘Rugby: Sky TV's Deal for All Blacks and Domestic Rugby Rights Worth $500m – Report.' New Zealand Herald. Available online at: https://www.nzherald.co.nz/sport/rugby-sky-tvs-deal-for-all-blacks-and-domestic-rugby-rights-worth-500m-report/6CXNBW2S67MWRTTO2DWDBOZJ3A/ (accessed February 4, 2021).

[B25] Horne J. (2018). Understanding the denial of abuses of human rights connected to sports mega-events. Leis. Stud. 37, 11–21. 10.1080/02614367.2017.1324512

[B26] Jackson S. J. Batty R. Scherer J. (2001). Transnational sport marketing at the global/local Nexus: the adidasification of the New Zealand all blacks Int. J. Sports Market. Sponsorship 3, 185–201. 10.1108/IJSMS-03-02-2001-B006

[B27] Jarvie G. Thornton J. Mackie H. (2018). Sport, Culture and Society. London, UK: Routledge.

[B28] Jhally S. (1989). “Cultural studies & the sports/media complex,” in Media, Sports & Society, ed Wenner L. (Newbury Park, CA: Sage), 70–93.

[B29] Johnson W. O. (1991). Sports in the year 2001: just by staying home, fans in the 21st century will become part of the action. Sports Illust. 75, 40–50.

[B30] Kane M. J. LaVoi N. M. Fink J. S. (2013). Exploring elite female athletes' interpretations of sport media images: a window into the construction of social identity and selling sex in women's sports. Commun. Sport 1, 269–298. 10.1177/2167479512473585

[B31] Keall C. (2021). Silver Lake: Why a Crew of Tech Geeks Created a Sports Empire. New Zealand Herald. Available online at: https://www.nzherald.co.nz/business/silver-lake-why-a-crew-of-tech-geeks-created-a-sports-empire/6CRLUT2ONCJK2RSDV7PZSMZHX4/ (accessed February 3, 2021).

[B32] Keles B. McCrae N. Grealish A. (2020). A systematic review: the influence of social media on depression, anxiety and psychological distress in adolescents. Int. J. Adolesc. Youth. 25, 79–93. 10.1080/02673843.2019.1590851

[B33] Kerr R. Kerr G. (2020). Promoting athlete welfare: a proposal for an international surveillance system. Sport Manage. Rev. 23, 95–103. 10.1016/j.smr.2019.05.005

[B34] Kihl L. A. Skinner J. Engelberg T. (2017). Corruption in sport: understanding the complexity of corruption. Eur. Sport Manage. Q. 17, 1–5. 10.1080/16184742.2016.1257553

[B35] Kuper S. (2006). Soccer Against the Enemy: How the World's Most Popular Sport Starts And Fuels Revolutions And Keeps Dictators in Power, 2nd Edn. New York, NY: Nation Books.

[B36] Laidlaw C. (1999). Rights of Passage: Beyond the New Zealand Identity Crisis. Auckland, New Zealand: Hodder Moa Beckett.

[B37] Lenskyj H. (2000). Inside the Olympic industry: Power, politics and activism. Albany, NY: SUNY Press.

[B38] Lewis J. (2013). Beyond Consumer Capitalism: Media and the Limits to Imagination. Cambridge, MA: Polity Press.

[B39] Lewis P. (2021). Why New Zealand Rugby Should take the Money and Run. New Zealand Herald. Available online at: https://www.nzherald.co.nz/sport/paul-lewis-why-new-zealand-rugby-should-take-the-money-and-run/MRRT523QPV7BIXTVGPBUP6FNBY/ (accessed February 6, 2021).

[B40] Maeckelbergh M. (2011). Doing is believing: prefiguration as strategic practice in the alterglobalization movement. Soc. Mov. Stud. 10, 1–20. 10.1080/14742837.2011.545223

[B41] Marcuse H. (1969). An Essay on Liberation. Available online at: https://www.marxists.org/reference/archive/marcuse/works/1969/essay-liberation.htm(accessed November 8, 2020).

[B42] Marx K. (2002). [1845]. Theses on Feuerbach. Transl.by Cyril Smith. Available online at: https://www.marxists.org/archive/marx/works/1845/theses/ (accessed July 20, 2021).

[B43] Miller T. (2018). Greenwashing sport. London: Routledge.

[B44] Napier L. (2020). New Zealand Rugby's Dire Warning to World Rugby Boss Bill Beaumont. New Zealand Herald. Available online at: https://www.nzherald.co.nz/sport/news/article.cfm?c_id=4&objectid=12329235 (accessed February 3, 2021).

[B45] Napier L. (2021). Exclusive: New Zealand Rugby field $465 million private investment offer from US tech giants Silver Lake. New Zealand Herald. Available online at: https://www.nzherald.co.nz/sport/exclusive-new-zealand-rugby-field-465-million-private-investment-offer-from-us-tech-giants-silver-lake/FSGCKRZ3LVO4Y2EKJ4XWIEMXH4/ (accessed February 3, 2021).

[B46] Orwell G. (1949). 1984. London: Secker & Warburg.

[B47] Parent S. Fortier K. (2018). Comprehensive overview of the problem of violence against athletes in sport. J. Sport Soc. Issues 42, 227–246. 10.1177/0193723518759448

[B48] Pavlidis A. Toffoletti K. Sanders K. (2020). “Pretty disgusted honestly”: exploring fans' affective responses on facebook to the modified rules of Australian Football league women's. J. Sport Soc. Issues 1–20. 10.1177/0193723520964969

[B49] Rowe D. (2020). Subjecting pandemic sport to a sociological procedure. J. Sociol. 56, 704–713. 10.1177/1440783320941284

[B50] Santos B. de S. (2006). The Rise of the Global Left: The World Social Forum and Beyond. London: Zed Books.

[B51] Scherer J. Jackson S. J. (2007). Sports advertising, cultural production and corporate nationalism at the global-local nexus: branding the New Zealand All Blacks. Sport Soc. 10, 268–284. 10.1080/17430430601147112

[B52] Scherer J. Jackson S. J. (2010). Sport, Globalisation and Corporate Nationalism: The New Cultural Economy of the New Zealand All Blacks. Oxford: eter Lang Publishers. 10.3726/978-3-0353-0000-0

[B53] Scherer J. Jackson S. J. (2013). The Contested Terrain of the New Zealand All Blacks: Rugby, Commerce, and Cultural Politics in the Age of Globalisation. Oxford: Peter Lang Publishers.

[B54] Schultz M. (2016). Debating futures: global trends, alternative visions, and public discourse. Int. Sociol. 31, 3–20. 10.1177/0268580915612941

[B55] Spaaij R. (2021). “Terrorism and sport,” in Research Handbook on Sports and Society, ed Pike E. (Cheltenham, UK: Edgar Publishing), 366–378.

[B56] Stanley A. (2021). How Women's Sport Can Prosper Post-Pandemic. Stuff. Available online at: https://www.stuff.co.nz/sport/women-in-sport/300228173/how-womens-sport-can-prosper-postpandemic (accessed February 12, 2021).

[B57] Stirling A. E. (2009). Definition and constituents of maltreatment in sport: establishing a conceptual framework for research practitioners. Br. J. Sports Med. 43, 1091–1099. 10.1136/bjsm.2008.051433 19028734

[B58] Tak M. Sam M. P. Jackson S. J. (2017). The politics of countermeasures against match-fixing in sport: a political sociology approach to policy instruments. Int. Rev. Sociol. Sport 53, 30–48. 10.1177/1012690216639748

[B59] Tak M. Sam M. P. Jackson S. J. (2018). The problems and causes of match-fixing: are legal sports betting regimes to blame? J. Criminol. Res. Pol. Pract. 4, 73–87. 10.1108/JCRPP-01-2018-0006

[B60] Taylor T. Toohey K. (2006). Impacts of terrorism related safety and security measures at a major sport event. Event Manage. 9, 119–209. 10.3727/152599506776771544

[B61] The Fan Project (2021a). Bringing Fans More Women's Sports. Available online at: https://thefanproject.co/ (accessed February 4, 2021).

[B62] The Fan Project (2021b). Why Sports Need the Fan Project. Available online at: https://thefanproject.co/about (accessed February 4, 2011).

[B63] Thorpe H. Toffoletti K. Bruce T. (2017). Sportswomen and social media: bringing third-wave feminism, postfeminism, and neoliberal feminism into conversation, J. Sport Soc. Issues 41, 359–383. 10.1177/0193723517730808

[B64] Toffler A. (1970). Future Shock. London: The Bodley Head.

[B65] Toffoletti K. Palmer C. (2019). Women and sport in Australia—new times? J. Aust. Stud. 43, 1–6. 10.1080/14443058.2019.1579081

[B66] Willson M. Tye M. Gorman S. Ely-Harper K. Creagh R. Leaver T.. (2018). Framing the women's AFL: contested spaces and emerging narratives of hope and opportunity for women in sport. Sport Soc. 21, 1704–1720. 10.1080/17430437.2017.1409727

[B67] Wilson B. Millington B. (2013). “Sport, ecological modernization, and the environment,” in A Companion to Sport, eds Andrews D. Carrington B. (Malden, MA: Blackwell Publishing), 129–142.

[B68] Winter C. J. (2020). Does time colonise intergenerational environmental justice theory? Environ. Polit. 29, 278–296. 10.1080/09644016.2019.1569745

[B69] Yates L. (2015). Rethinking prefiguration: alternatives, micropolitics and goals in social movements. Soc. Mov. Stud. 14, 1–21. 10.1080/14742837.2013.870883

[B70] Young K. (2019). Sport, Violence and Society. Oxford: Routledge. 10.4324/9781315737089

[B71] Zuboff S. (2019). Surveillance capitalism and the challenge of collective action. New Labor Forum 28, 10–29. 10.1177/1095796018819461

